# Fasting blood glucose, glycaemic control and prostate cancer risk in the Finnish Randomized Study of Screening for Prostate Cancer

**DOI:** 10.1038/s41416-018-0055-4

**Published:** 2018-03-22

**Authors:** Teemu J. Murtola, Ville JY Vihervuori, Jorma Lahtela, Kirsi Talala, Kimmo Taari, Teuvo LJ Tammela, Anssi Auvinen

**Affiliations:** 1 Faculty of Medicine and life Sciences, University of Tampere, Tampere, Finland; 2 Department of Urology, Tampere University Hospital, Tampere, Finland; 3 Department of Internal Medicine, Tampere University Hospital, Tampere, Finland; 4 Finnish Cancer Registry, Helsinki, Finland; 5 Department of Urology, Helsinki University and Helsinki University Hospital, Helsinki, Finland; 6 Faculty of Social Sciences, University of Tampere, Tampere, Finland

**Keywords:** Risk factors, Prostate cancer

## Abstract

**Background:**

Diabetic men have lowered overall risk of prostate cancer (PCa), but the role of hyperglycaemia is unclear. In this cohort study, we estimated PCa risk among men with diabetic fasting blood glucose level.

**Methods:**

Participants of the Finnish Randomized Study of Screening for Prostate Cancer (FinRSPC) were linked to laboratory database for information on glucose measurements since 1978. The data were available for 17,860 men. Based on the average yearly level, the men were categorised as normoglycaemic, prediabetic, or diabetic. Median follow-up was 14.7 years. Multivariable-adjusted Cox regression was used to calculate hazard ratios (HRs) and 95% confidence intervals (95% CIs) for prostate cancer overall and separately by Gleason grade and metastatic stage.

**Results:**

In total 1,663 PCa cases were diagnosed. Compared to normoglycaemic men, those men with diabetic blood glucose level had increased risk of PCa (HR 1.52; 95% CI 1.31–1.75). The risk increase was observed for all tumour grades, and persisted for a decade afterwards. Antidiabetic drug use removed the risk association. Limitations include absence of information on lifestyle factors and limited information on BMI.

**Conclusions:**

Untreated diabetic fasting blood glucose level may be a prostate cancer risk factor.

## Introduction

The relationship between established type 2 diabetes mellitus (DM) and different types of cancer has been investigated broadly. Studies have shown that people with DM have lower risk of developing prostate cancer (PCa) compared with non-diabetics.^1, 2^ This may be due to altered hormone milieu in diabetic men or lower prostate-specific antigen (PSA) leading to less prostate biopsies due to elevated PSA. However, it is not clear whether this concerns also poorly differentiated PCa, as some studies have suggested that the risk could be elevated.^3^ Glucose metabolism may have an independent role in prostate cancer development and progression.^4, 5^ There is evidence that hyperglycaemia and poor glycaemic control and the related hyperinsulinaemia could be the possible links.^6–8^ Yet there are very few studies concentrating on connections between PCa and blood glucose levels and glycaemic control, and the results from available studies are conflicting. Some have reported increased risk of advanced or aggressive PCa among men with higher glucose levels,^9^ while other studies have reported a protective association between hyperglycaemia and PCa.^4, 10^ Indirect support for the role of hyperglycaemia as PCa risk factor comes from studies reporting lowered risk among men using antidiabetic drugs, particularly metformin.^11^Author surnames have been highlighted - please check these carefully and indicate if the first name or surname have been marked up incorrectly. Please note that this will affect indexing of your article, such as in PubMed.

In this study, we investigated the associations between fasting blood glucose and glycaemic control (glycated haemoglobin, HbA1c) and PCa risk overall and by Gleason grade and tumour stage in a cohort of men participating in The Finnish Randomized Study of Screening for Prostate Cancer (FinRSPC),^12^ the largest component of European Randomized Study of Screening for Prostate Cancer (ERSPC).^13^ The main goal of FinRSPC and ERSPC studies was to find out if systematic screening can decrease prostate cancer mortality. A significant reduction in PCa mortality was observed in the ERSPC study, though with overdiagnosis of well-differentiated prostate cancer. In the FinRSPC study the reduction in PCa mortality was smaller and not statistically significant.^12^

## Materials and methods

### Study cohort

The Finnish Randomized Study of Screening for Prostate Cancer (FinRSPC) is the largest component of the multinational European Study of Prostate Cancer Screening (ERSPC).^13^ In the FinRSPC, all men aged 55–67 from Tampere and Helsinki residential areas were identified between 1996 and 1999. After exclusion of prevalent prostate cancer cases identified from national Finnish Cancer Registry, a total of 80,144 men were randomly assigned either to be screened with PSA at four-year intervals (the screening arm, 31,866 men) or to control arm with no intervention and followed through national registries (48,278 men). The screening finished in 2008, but the follow-up for cancer cases in this study extended until the end of 2014. The available information on prostate cancers were obtained trough study database or through The Finnish Cancer Registry for men in the control arm. The information included date of diagnosis, tumour Gleason score, and tumour stage at diagnosis, recorded in this study as non-metastatic (all M0 cases) and metastatic (all M1 cases).

The Finnish Cancer Registry is a nationwide registry collecting information of cancer diagnoses from all Finnish health care units and pathology laboratories through mandatory notifications. The registry covers more than 99% of all cancer cases diagnosed in Finland.^14^

To obtain information on BMI the participants of the third screening round during 2004–2008 were mailed surveys asking height and weight along with the screening invitations.^15^ This information was available for 11,698 men in the FinRSPC cohort.

The study cohort was linked to a nationwide prescription database of the Social Insurance Institution (SII) of Finland. The SII is a governmental agency providing reimbursements for the cost of physician-prescribed drugs to all Finnish citizens. From this database we obtained information on usage of prescribed antidiabetic drugs, antihypertensive drugs, statins, NSAIDs, and aspirin during 1995–2009. Information on over-the-counter drug purchases or on drugs administered during hospital inpatient periods was not available.

### Information on blood glucose level and glycated haemoglobin

The information on results from each measurement of fasting plasma or blood glucose level and glycated haemoglobin (HbA1c) performed at the Pirkanmaa region were obtained from the Fimlab database and linked to the study cohort by using individual identifier number. The data were available from 1978 for fasting blood glucose level and from 1991 for HbA1c. Information considering indication of blood glucose measurements was not available, but it is most likely that these measurements are a mix of standard health checks, age-dependent health checks, and control measurements of diabetic men.

Fimlab Laboratories is one of the leading laboratory companies in Finland.^16^ Fimlab provides laboratory services, education, and research in Pirkanmaa, Central Finland, and Tavastia regions. A major part of laboratory measurements in the Pirkanmaa region are done in Fimlab laboratories. Approximately 25% of the FinRSPC participants lived at Pirkanmaa region at baseline.

The study population of the FinRSPC study was linked to the Fimlab database to identify men with at least one available measurement of blood glucose and/or HbA1c during 1978–2015. The information on fasting glucose levels and/or HbA1c levels were available for 19,263 men, thus forming the cohort of this study (Supplementary figure). The information on fasting glucose levels or HbA1c levels were available for 17,860 and 11,736 men, respectively. Each measured fasting glucose level was divided into three categories: normoglycaemic if fasting glucose level was under or equal to 6.0 mmol/l measured from plasma or under or equal to 5.5 mmol/l measured from blood; pre-diabetic if glucose level was between 6.0 and 7.0 mmol/l (plasma) or between 5.6 and 6.1 mmol/l (blood), and diabetic if glucose level was over 7.0 mmol/l (plasma) or over 6.1 mol/l (blood). The HbA1c levels were also divided into three categories: normoglycaemic if HbA1c was under 39 mmol/mol or under 5.7%, prediabetic if HbA1c was between 39 and 47 mmol/mol or between 5.7 and 6.4%, and diabetic if HbA1c level was over 47 mmol/mol or over 6.4%.

### Statistical analysis

We used Cox proportional hazards regression to estimate hazard ratios (HRs) and 95% confidence intervals (CIs) for overall risk of prostate cancer by fasting glucose and HbA1c levels. Analyses for Gleason 6 or less, Gleason 7–10, non-metastatic and metastatic tumours were performed separately. These separate analyses were carried out in the same way as the overall analysis, but we only included those PCa cases that met the specific criteria, and calculated HRs between different groups for that cancer type. The follow-up commenced from the FinRSPC randomisation (baseline) and ended at the date of death, diagnosis of prostate cancer, emigration, or end of the follow-up time (31 December 2014), whichever came first. The time axis was years and month since the baseline. The model was adjusted for age only (age-adjusted analysis) and further for the FinRSPC study arm and the use of antihypertensive drugs, cholesterol-lowering drugs, NSAIDs, and aspirin (multivariable-adjusted model).

For each calendar year, we calculated the average yearly fasting glucose and HbA1c levels based on all measurements within that year. Fasting glucose level and HbA1c were analysed as time-dependent variables, updated separately for each year after the baseline. All men who did not have any glucose or HbA1c measurement available in certain year was assigned to separate missing-category for that year.

We further calculated the average blood glucose levels both before and after initiation of antidiabetic drug use, i.e., the first recorded purchase of any antidiabetic drug. Fasting glucose level before and after antidiabetic drug use was analysed as time-independent variables.

The association between fasting glucose level and prostate cancer risk in subgroups was evaluated by repeating the analyses after stratifying the study population by background variables. Subgroup analyses were stratified by use of drugs that are associated with metabolic syndrome and chronic inflammation. We tested effect modification by adding an interaction term between blood glucose level and the effect modifier into the Cox regression model. The interaction term was considered statistically significant if the *p*-value was ≤0.05.

The temporal association between fasting glucose level and prostate cancer risk was evaluated by performing separate lag time analysis for measurements done five, ten, fifteen, and twenty years earlier.

We used IBM SPSS statistics software (version 23) to perform Cox regression analyses. All *p*-values are two-sided.

### Ethics approval of the study

The study has been approved by the ethics committee of the Pirkanmaa Hospital District, decision number R10167.

## Results

### Population characteristics

Of the 17,860 men, 8,481 were normoglycaemic, 5,812 were at the pre-diabetic range, and 3,567 men were at the diabetic range of fasting glucose (Table 1). During the median follow-up of 14.7 years after the baseline, a total of 1,663 prostate cancers were diagnosed in the study cohort, 808 in the normoglycaemic group, 454 in the pre-diabetic group, and 401 in the diabetic group. Median BMI in the diabetic group was non-significantly higher compared to the normoglycaemic group.

**Table 1:** Population characteristics

	Average fasting blood glucose level during follow-up		
	Normoglycaemic	Pre-diabetic	Diabetic
*n* of men	8,481	5,812	3,567
FinRSPC study arm			
Screening; *n* (%)	3,154 (37.2)	2,253 (38.8)	1,398 (39.2)
Control; *n* (%)	5,327 (62.8)	3,559 (61.2)	2,169 (60.8)
Median (IQR) follow-up	17.0 (15.1–18.0)	16.3(13.8–18.0)	15.3(7.1–17.0)
*n* of PCa cases	808	454	401
Total follow-up time (person years)	130,811	87,400	43,779
PCa incidence/1000 person years	6.18	5.19	9.16
Gleason score; *n* (%)			
6 or less	409 (50.6)	215 (47.4)	203 (50.6)
7–10	384 (47.5)	230 (50.7)	189 (47.1)
Missing	15 (1.9)	9 (2.0)	9 (2.2)
Tumour stage; *n* (%)			
Localised	759 (93.9)	410 (90.3)	374 (93.3)
Metastatic	48 (5.9)	44 (9.7)	25 (6.2)
Missing	1 (0.1)	0 (0.0)	2 (0.5)
Median (IQR) PSA^a^ in			
1st screening round	1.02 (0.62–1.76)	0.97 (0.61–1.69)	1.01 (0.58–2.06)
*P* for difference^b^	Ref	0.21	0.47
2nd screening round	1.30 (0.77–2.28)	1.23 (0.72–2.26)	1.16 (0.66–2.31)
*P* for difference^b^	Ref	0.13	0.01
3rd screening round	1.43 (0.81–2.49)	1.39 (0.77–2.41)	1.19 (0.67–2.24))
*P* for difference^b^	Ref	0.11	<0.001
Median (IQR) BMI	26.0 (24.2–28.3)	27.7 (25.3–30.6)	28.6 (26.2–31.6)
Antidiabetic medication use; *n* (%)^c^	207 (2.4)	1,370 (23.6)	2,376 (66.6)
*P*for difference^d^	Ref	<0.001	<0.001
Statin use; *n* (%)	3,291 (38.8)	2,708 (46.6)	1,851 (51.1)
*P* for difference^d^	Ref	<0.001	<0.001
Antihypertensive drug use; *n* (%)	5,481 (64.6)	4,387 (75.5)	2,917 (81.8)
*P* for difference^d^	Ref	<0.001	<0.001
NSAID use; *n* (%)	6,845 (80.7)	4,692 (80.7)	2,749 (77.0)
*P* for difference^d^	Ref	0.98	<0.001
ASA use; *n* (%)	1,536 (18.1)	1,159 (19.9)	715 (20.0)
*P* for difference^d^	Ref	0.006	0.013
5α-reductase inhibitor use; *n* (%)	1,253 (14.8)	724 (12.5)	384 (10.8)
*P* for difference^d^	Ref	<0.001	<0.001

Study cohort of 17,860 men from the Finnish Randomized Study of Screening for Prostate Cancer with at least one fasting blood/plasma glucose measurement available during 1978–2014.

^a^All PSA values measured are from men in the screening arm of the study.

^b^*P* for difference compared to the normoglycaemic group. Calculated with chi-square test.

^c^Categorisation based on average blood glucose level, thus also users of antidiabetic drugs may be included in the normoglycaemic group if they have good blood glucose control.

^d^*P* for difference compared to the normoglycaemic group. Calculated with Mann–Whitney *U*-test

The use of cholesterol-lowering drugs, antihypertensive drugs, and aspirin was more common in the diabetic group compared to the normoglycenic or pre-diabetic group. However, use of NSAIDs and 5α-reductase inhibitors was less common in the diabetic group (Table 1).

Median PSA was lower among diabetic men in all three FinRSPC screening rounds compared to normoglycaemic men (Table 1).

### Fasting blood glucose, HbA1c, and prostate cancer risk

Compared to the normoglycaemic men, overall PCa risk was elevated in diabetic, but not in pre-diabetic men; multivariable adjusted HR 1.52; 95% CI 1.31–1.75 (Table 2). The risk was similarly elevated regardless of tumour Gleason score. However, the risk elevation was limited to non-metastatic tumours; fasting blood glucose level was not associated with risk of metastatic prostate cancer (Table 2). Association between HbA1c levels and PCa risk was not statistically significant. However, the number of men in these analyses was low.

**Table 2:** Prostate cancer risk by average fasting blood glucose and HbA1c level

		Prostate cancer risk									
		Overall		Gleason 6 or less		Gleason 7–10		Localised PCa		Metastatic	
	*n* of men/PCa cases	HR (95% CI)_age-adjusted_	HR (95% CI)_multivar.-adjusted_^a^	HR (95% CI)_age-adjusted_	HR (95% CI)_multivar.-adjusted_^a^	HR (95% CI)_age-adjusted_	HR (95% CI)_multivar.-adjusted_^a^	HR (95% CI)_age-adjusted_	HR (95% CI)_multivar.-adjusted_^a^	HR (95% CI)_age-adjusted_	HR (95% CI)_multivar.-adjusted_^a^
Fasting blood glucose level											
Normal	8,481/808	Ref	Ref	Ref	Ref	Ref	Ref	Ref	Ref	Ref	Ref
Pre-diabetic	5,812/454	1.07 (0.99–1.23)	1.09 (0.95–1.26)	1.03 (0.84–1.27)	1.08 (0.95–1.26)	1.09 (0.89–1.34)	1.10 (0.90–1.36)	1.10 (0.94–1.27)	1.13 (0.97–1.31)	0.75 (0.43–1.31)	0.74 (0.42–1.30)
Diabetic	3,567/401	1.36 (1.19–1.56)	1.52 (1.31–1.75)	1.26 (1.04–1.53)	1.48 (1.21–1.81)	1.38 (1–13–1.70)	1.46 (1.18–1.81)	1.39 (1.21–1.61)	1.57 (1.35–1.82)	0.99 (0.58–1.69)	0.94 (0.54–1.64)
HbA1c level											
Normal	3,978/431	Ref	Ref	Ref	Ref	Ref	Ref	Ref	Ref	Ref	Ref
Pre-diabetic	4,342/338	1.13 (0.88–1.44)	1.15 (0.90–1.47)	1.07 (0.75–1.54)	1.11 (0.77–1.59)	1.17 (0.83–1.64)	1.18 (0.84–1.66)	1.13 (0.87–1.46)	1.16 (0.89–1.50)	1.05 (0.42–2.62)	1.08 (0.43–2.70)
Diabetic	2,880/323	0.95 (0.73–1.23)	1.04 (0.80–1.35)	0.77 (0.53–1.13)	0.89 (0.60–1.31)	1.08 (0.75–1.53)	1.11 (0.77–1.59)	0.87 (0.67–1.15)	0.96 (0.73–1.26)	1.77 (0.73–4.30)	1.83 (0.74–4.52)

Study cohort of 17,860 men from the Finnish Randomized Study of Screening for Prostate Cancer.

^a^Multivariable-adjusted analyses adjusted with age, the FinRSPC study arm and use of antihypertensive drugs, statins, NSAIDs or aspirin

### Effect modification by background variables

**Fig. 1:**
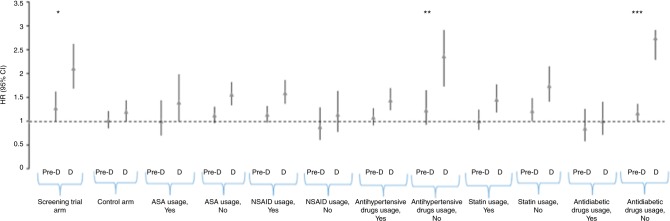
Association between overall prostate cancer risk and blood glucose level in subgroup analysis. Study cohort of 17,860 men from the Finnish Randomized Study of Screening for Prostate Cancer. Pre-D pre-diabetic, D diabetic blood glucose level. *P for interaction = 0.229 for pre-diabetic, *p* < 0.001 for diabetic men. **P for interaction = 0.435 for pre-diabetic, *p* = 0.018 for diabetic men. ***P for interaction = 0.153 for pre-diabetic, *p* < 0.001 for diabetic men. NSAID non-steroidal anti-inflammatory drugs, ASA acetylsalicylic acid

The connection between fasting glucose level and PCa risk was affected by several background variables (Fig. 1). Diabetic blood glucose level was associated with increased risk in the FinRSPC screening arm, but not in the control arm (*p* for interaction <0.001). The risk association was also modified by the use of antihypertensive drugs; the association was weaker in users of these drugs (*p* for interaction 0.018). Further, use of antidiabetic drugs generally and in separate analysis metformin use abolished the risk association (*p* for interaction <0.001). No clear effect modification by 5-ARI use was observed, either (*p* for interaction 0.78).

**Fig. 2:**
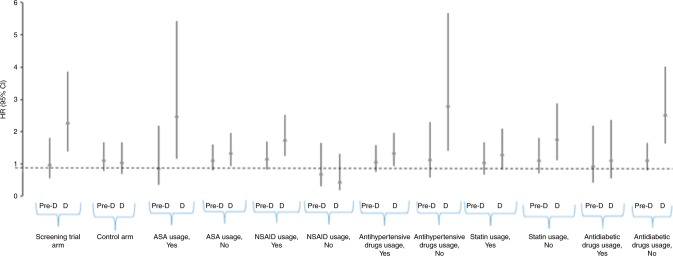
Association between Gleason 8–10 prostate cancer risk and blood glucose level in subgroup analysis. Study cohort of 17,860 men from the Finnish Randomized Study of Screening for Prostate Cancer. Pre-D pre-diabetic, D diabetic blood glucose level. NSAID non-steroidal anti-inflammatory drugs, ASA acetylsalicylic acid

Similar risk modifications were observed also for Gleason score 8–10 tumours (Fig. 2). However, the interactions were not statistically significant in this subgroup.

### Role of change in fasting glucose levels after initiation of antidiabetic drug use

Changes in fasting blood glucose levels after the initiation of antidiabetic drug use were not associated with changes in prostate cancer risk (Table 3). Similarly, no change in prostate cancer risk was observed by changes in fasting glucose levels after initiation of metformin use.

**Table 3:** Impact of change in fasting blood glucose level after initiation of antidiabetic drug use

	Prostate cancer risk					
		Overall	Gleason 6 or less	Gleason 7–10	Localised PCa	Metastatic
	*n* of men/PCa cases	HR (95% CI)_multivar.-adjusted_^a^	HR (95% CI)_multivar.-adjusted_^a^	HR (95% CI)_multivar.-adjusted_^a^	HR (95% CI)_multivar.-adjusted_	HR (95% CI)_multivar.-adjusted_^a^
Change in fasting blood glucose level						
No change	427/43	Ref	Ref	Ref	Ref	Ref
Decrease	873/45	0.82 (0.49–1.36)	0.85 (0.39–1.84)	0.74 (0.37–1.47)	0.67 (0.38–1.20)	2.87 (0.87–9.50)
Increased	933/41	0.83 (0.55–1.26)	1.07 (0.59–1.93)	0.62 (0.34–1.13)	0.85 (0.54–1.29)	0.92 (0.23–3.68)

Study cohort of 17,860 men from the Finnish Randomized Study of Screening for Prostate Cancer.

^a^Multivariable-adjusted analyses adjusted with age, the FinRSPC study arm and use of antihypertensive drugs, statins, NSAIDs, or aspirin

### Long-term effects of fasting glucose levels

Blood glucose level measured 10 years earlier was borderline significantly associated with elevated PCa risk as in our main analysis; diabetic fasting glucose level was associated with increased risk of non-metastatic tumours and Gleason 6 or less tumour (Table 4). Similar, albeit non-significant risk increase were observed also with 15-year lag time. However, no risk associations were observed for the blood glucose levels measured two decades earlier.

**Table 4:** Association between the fasting blood glucose level 5, 10, 15, and 20 years earlier and prostate cancer risk

	Prostate cancer risk				
	Overall	Gleason 6 or less	Gleason 7–10	Localised PCa	Metastatic
	HR (95% CI) _multivar.-adjusted’_	HR (95% CI) _multivar.-adjusted’_	HR (95% CI) _multivar.-adjusted’_	HR (95% CI) _multivar.-adjusted’_	HR (95% CI) _multivar.-adjusted’_
Average fasting blood glucose level 5 years earlier					
Normal	Ref	Ref	Ref	Ref	Ref
Pre-diabetic	0.91 (0.70–1.17)	0.71 (0.47–1.07)	1.09 (0.78–1.52)	0.89 (0.68–1.17)	1.08 (0.47–2.50)
Diabetic	1.00 (0.78–1.27)	0.63 (0.42–0.94)	1.37 (1.01–1.88)	0.96 (0.74–1.17)	1.25 (0.56–2.76)
Average fasting blood glucose level 10 years earlier					
Normal	Ref	Ref	Ref	Ref	Ref
Pre-diabetic	1.27 (0.78–1.90)	0.63 (0.26–1.52)	1.70 (0.98–2.92)	1.16 (0.73–1.86)	1.97 (0.44–8.82)
Diabetic	1.34 (0.93–1.93)	1.62 (0.97–2.69)	1.31 (0.67–1.91)	1.41 (0.97–2.04)	0.38 (0.04–3.42)
Average fasting blood glucose level 15 years earlier					
Normal	Ref	Ref	Ref	Ref	Ref
Pre-diabetic	0.81 (0.34–1.92)	0.62 (0.14–2.68)	0.93 (0.32–2.76)	0.79 (0.31–2.05)	1.07 (0.12–9.59)
Diabetic	1.03 (0.58–1.82)	1.52 (0.70–3.27)	0.58 (0.23–1.50)	1.22 (0.68–2.20)	—
Average fasting blood glucose level 20 years earlier					
Normal	Ref	Ref	Ref	Ref	Ref
Pre-diabetic	0.30 (0.04–2.20)	0.65 (0.08–5.07)	—	0.32 (0.04–2.35)	—
Diabetic	0.97 (0.41–2.32)	—	1.78 (0.68–4.67)	0.90 (0.36–2.26)	1.51 (0.15–37.8)

Study cohort of 17,860 men from the Finnish Randomized Study of Screening for Prostate Cancer.

Multivariable-adjusted analyses adjusted with age, the FinRSPC study arm and use of antihypertensive drugs, statins, NSAIDs, or aspirin

## Discussion

In this study we observed an association between fasting blood glucose level and elevated prostate cancer risk. This association was more noticeable in the screening arm, and concerned both poorly and well-differentiated cancers. Previous studies suggest that DM and use of antidiabetic drugs are associated with lowered overall prostate cancer risk, while the risk of high-grade tumours may be elevated.^17–20^ Our findings do not support the risk lowering association for diabetes as a condition, as diabetic fasting blood glucose levels were associated with increased, not lowered prostate cancer risk. This supports the role of hyperglycaemia as a prostate cancer risk factor. Nevertheless, use of antidiabetic drugs abolished the risk increase observed with diabetic fasting blood glucose level. Therefore, our study indirectly supports anticancer effects of antidiabetic drugs, but the mechanism of action is probably unrelated to blood glucose levels.

Some previous studies have reported association between diabetes and lowered PSA compared to non-diabetic men.^21–23^ Concordantly, median PSA was lower also in our study. This would presumably lead to lower incidence of PCa, as fewer prostate biopsies would be performed due to PSA elevation. In a setting of prostate cancer screening this would mean lower PCa incidence among diabetic men especially in the FinRSPC screening arm. In contrast, we observed a stronger association with increased PCa risk in the FinRSPC screening arm, which suggests that prostate cancer incidence among diabetic men is higher compared to non-diabetic men even if the median PSA is lower. The risk elevation seems to apply to all tumour grades as the risk elevation was observed also for high-grade tumours.

A previous meta-analysis of 14 epidemiological studies found increased risk of several cancer types for elevated HbA1c, excluding prostate cancer.^4^ Concordantly, we did not observe risk associations with HbA1c, only with fasting blood glucose levels.

Another previous study has examined the association between blood glucose and prostate cancer risk.^24^ In this case-cohort study of 500 participants insulin concentrations and glucose concentrations were determined from a single overnight fasting serum sample 5 to 12 years before prostate cancer diagnosis. The study suggests an association between PCa risk and elevated fasting serum insulin, but not with elevated glucose levels. In contrast to our study, the observed blood glucose levels were mainly in the normal range, and no diabetic blood glucose levels were observed. Thus our results are in concordance with this previous study, as we did not observe any risk increase even in men with pre-diabetic blood glucose level, only among men at diabetic glucose level.

The significance of blood glucose as PCa risk factor is supported by our earlier findings in which we found a connection between changes in genes associated to glucose metabolism and PCa risk.^5^

Previously reported lower PCa risk among men with diabetes could be explained by use of antidiabetic medication; for example lower PCa risk has been reported among metformin users.^25–28^ In this study the use of antidiabetic drugs abolished the observed association between high blood glucose levels and elevated risk of prostate cancer. This supports that the treatment of diabetes could remove the risk increase associated with hyperglycaemia. However, the changes in blood glucose level after initiation of antidiabetic drug use during the follow-up was not connected to PCa risk, which suggests that the possible antitumour effects of antidiabetic drugs are mediated through mechanisms other than blood glucose control. However, confirmation will be needed from other studies as confidence intervals were relatively wide in our risk estimates.

The association between blood glucose level and PCa risk is likely to be long-term, because the elevated risk was observable also for the fasting blood glucose level measured a decade earlier. This suggests that diabetic glucose level could increase the risk of initiation of prostate cancer development. To our knowledge this is the first study to examine long-term associations between fasting blood glucose and prostate cancer risk.

However, we did not observe risk associations by HbA1c level, which reflects long-term glucose balance. HbA1c results were available for fewer participants than were fasting glucose measurements, thus these analyses were limited by lowered statistical power. Nevertheless, this difference between the risk associations between HbA1c and fasting glucose suggests that the interaction between blood glucose, insulin and prostate cancer may be complex.

Strengths of our study include large population-based cohort and information on blood glucose measurements starting from the 1980’s. We were also able to evaluate the effect of antidiabetic drug on the risk association through comprehensive national prescription database free of recall bias.

The study also has some limitations. We had no information on lifestyle factors, such as smoking, diet, or physical activity, which could possibly affect prostate cancer risk and cause confounding.^29–31^ The available information on BMI was also limited. Because of these factors, residual confounding is possible. Also, because we had no information on the indication of blood glucose measurements, selection bias is possible affecting the generalisability of our results. However, it does not limit our comparisons by blood glucose level because all men with blood glucose measurements available are prone to the same selection. Our study population is almost entirely Caucasian due to homogeneity of the Finnish population; thus our results may not be generalisable to other ethnicities.

In conclusion, diabetic fasting blood glucose level is associated with elevated PCa risk in a population-based cohort of Finnish men, especially in the setting of systematic PSA-based screening. The risk association is long-term, observed a decade in advance of the diagnosis. Use of antidiabetic drugs removes the risk association, supporting the risk lowering effect of these drugs.

## Electronic supplementary material

Supplementary Figure

## Availability of data and material:

Data used in this study are available at request from the authors.
