# Supplementary material for: Fasting blood glucose, glycaemic control and prostate cancer risk in the Finnish Randomized Study of Screening for Prostate Cancer
Source: Br J Cancer. 2018 Mar 22;118(9):1248–54. doi: 10.1038/s41416-018-0055-4 (PMC5943324; doi:10.1038/s41416-018-0055-4)
Supplement: Supplementary file 1 — Supplementary Figure [file 41416_2018_55_MOESM1_ESM.docx]

Men participating in the FinRSPC study (80,144)

Study population linked to the Fimlab database.

All men who had at least one fasting blood / plasma glucose and / or HbA1C-measurement taken (19,263)

- Screening arm: 7,359

- Control arm: 11,868

Men with HbA1C-measurements (11,736)

- Screening arm: 4,491

- Control arm: 7,245

Men with glucose measurements (17,860)

- Screening arm: 6,085
- Control arm: 11,055

**Supplementary Figure.** Flow chart for forming of the study population. Study cohort of 17,860 men from the Finnish Randomized Study of Screening for Prostate Cancer with at least one fasting blood/plasma glucose measurement available during 1978-2014.
